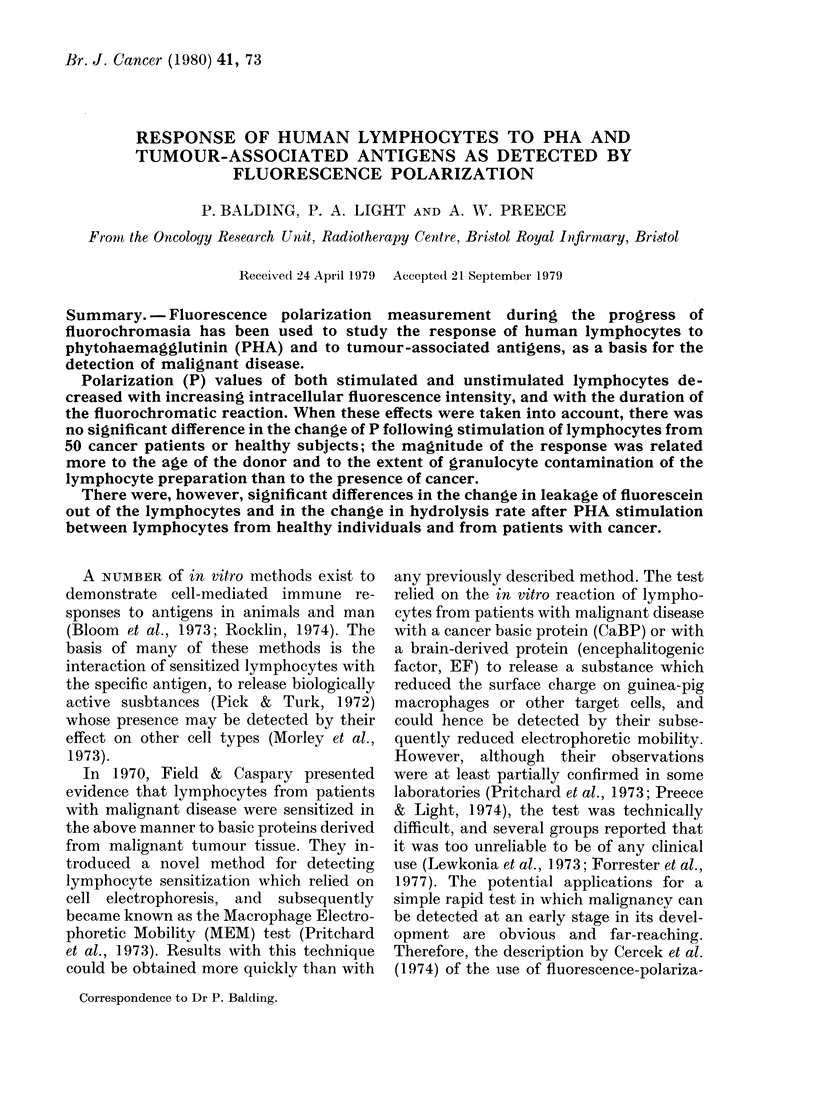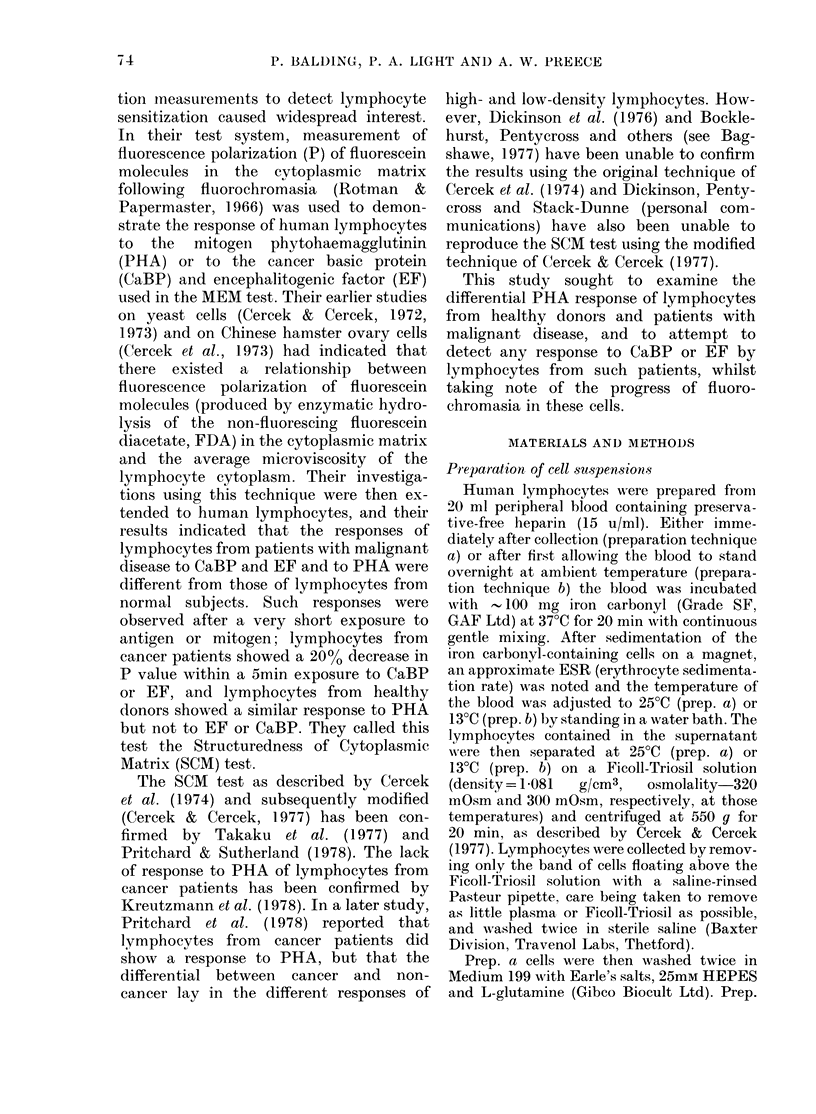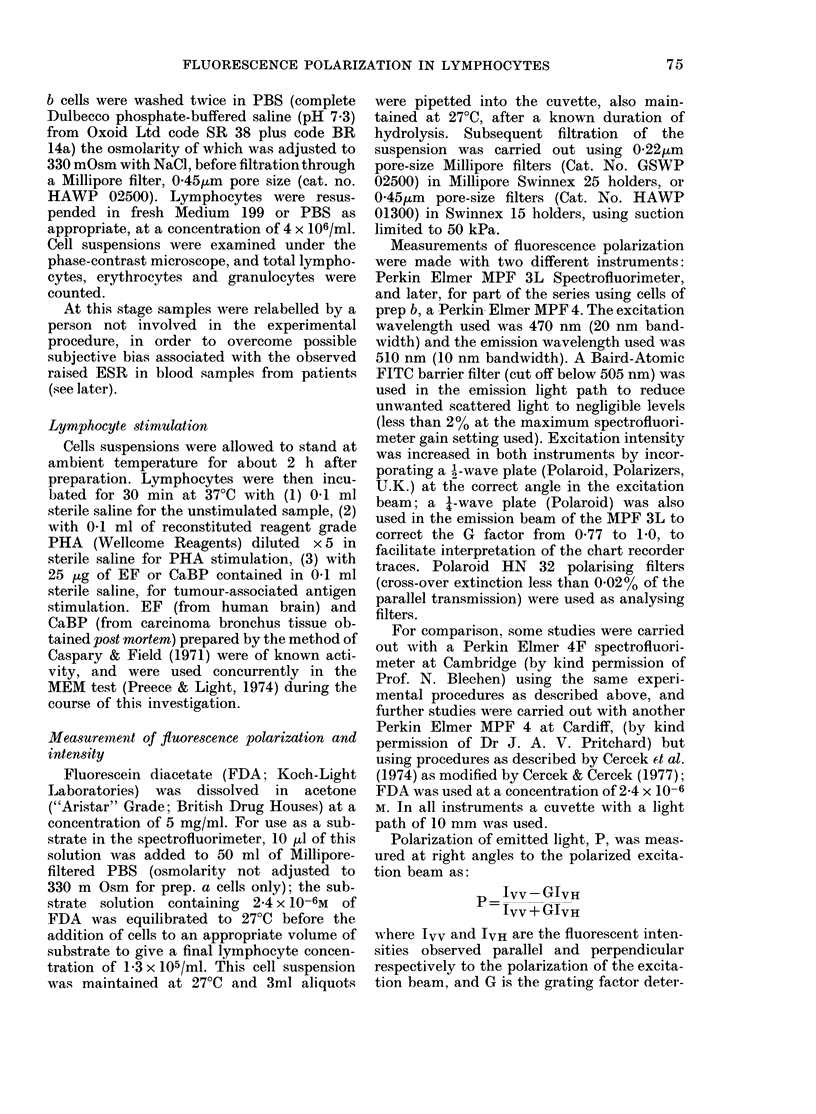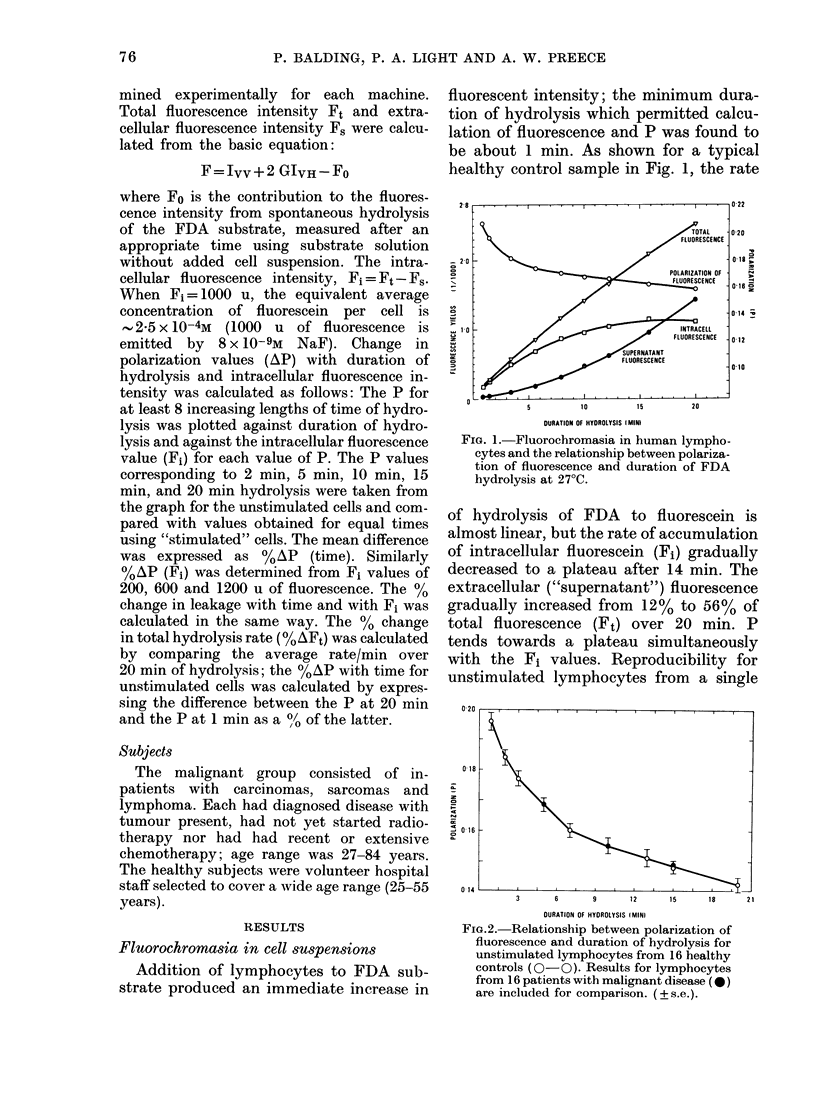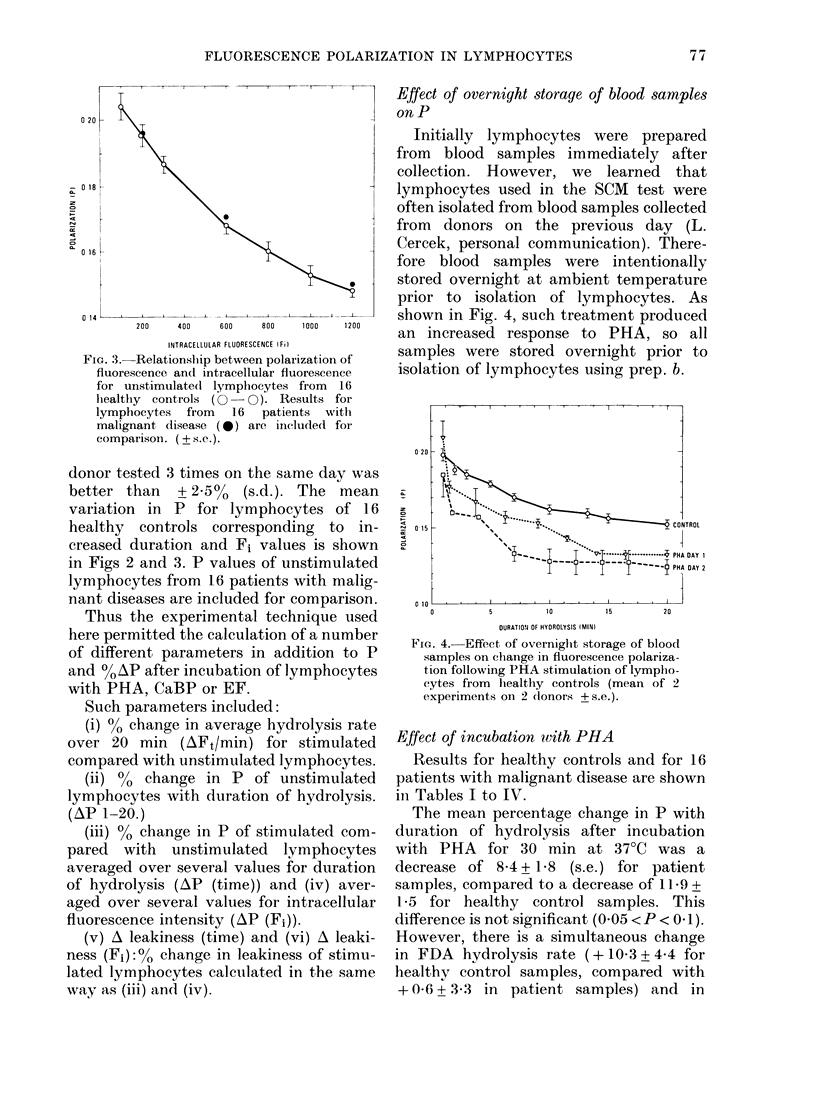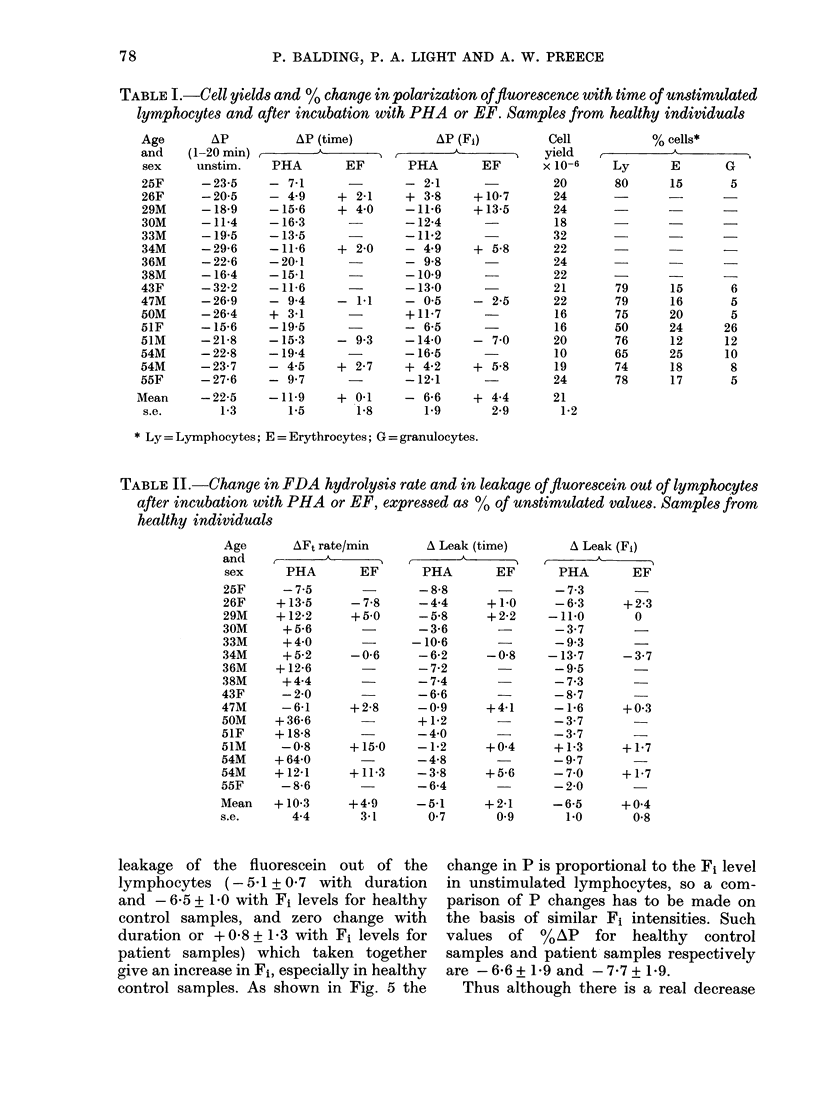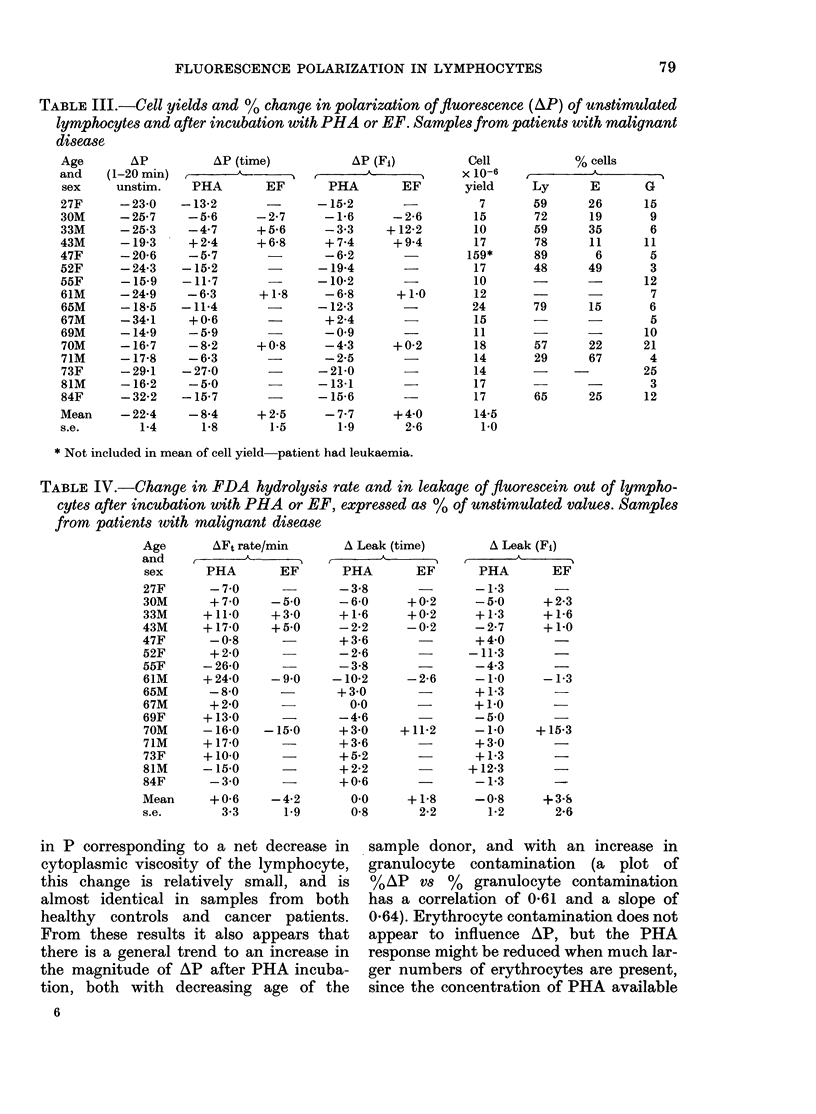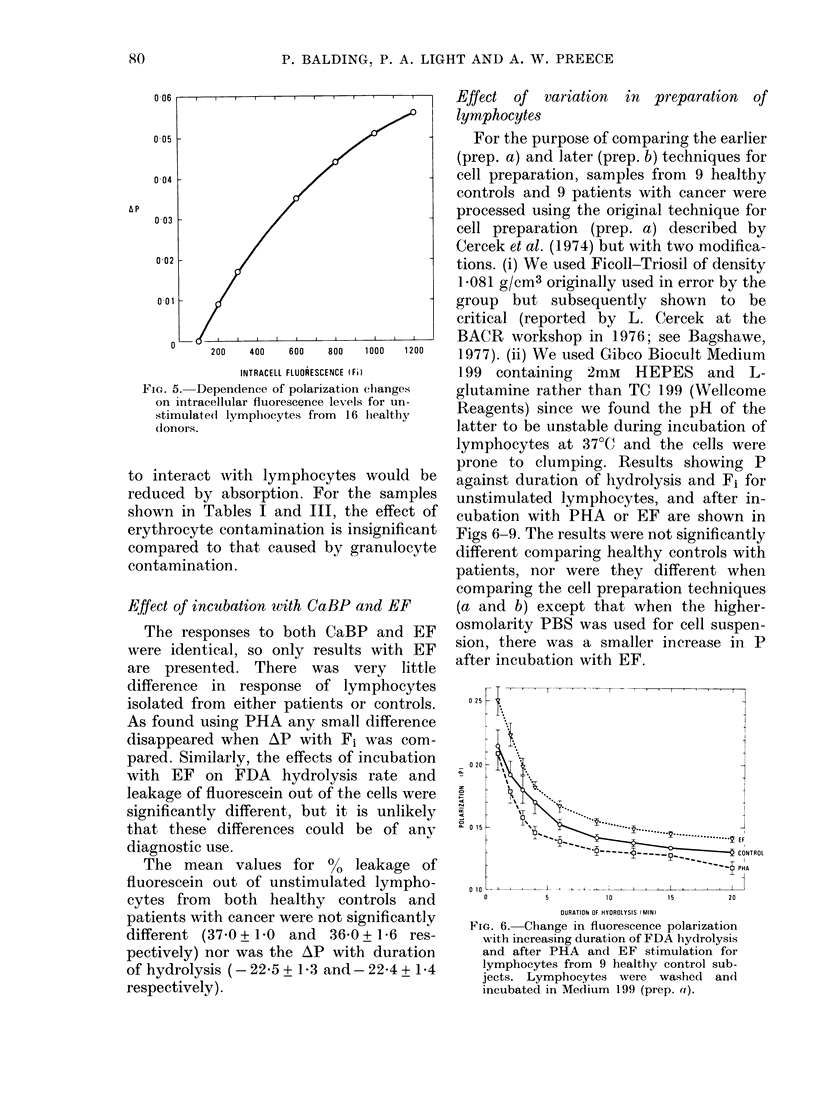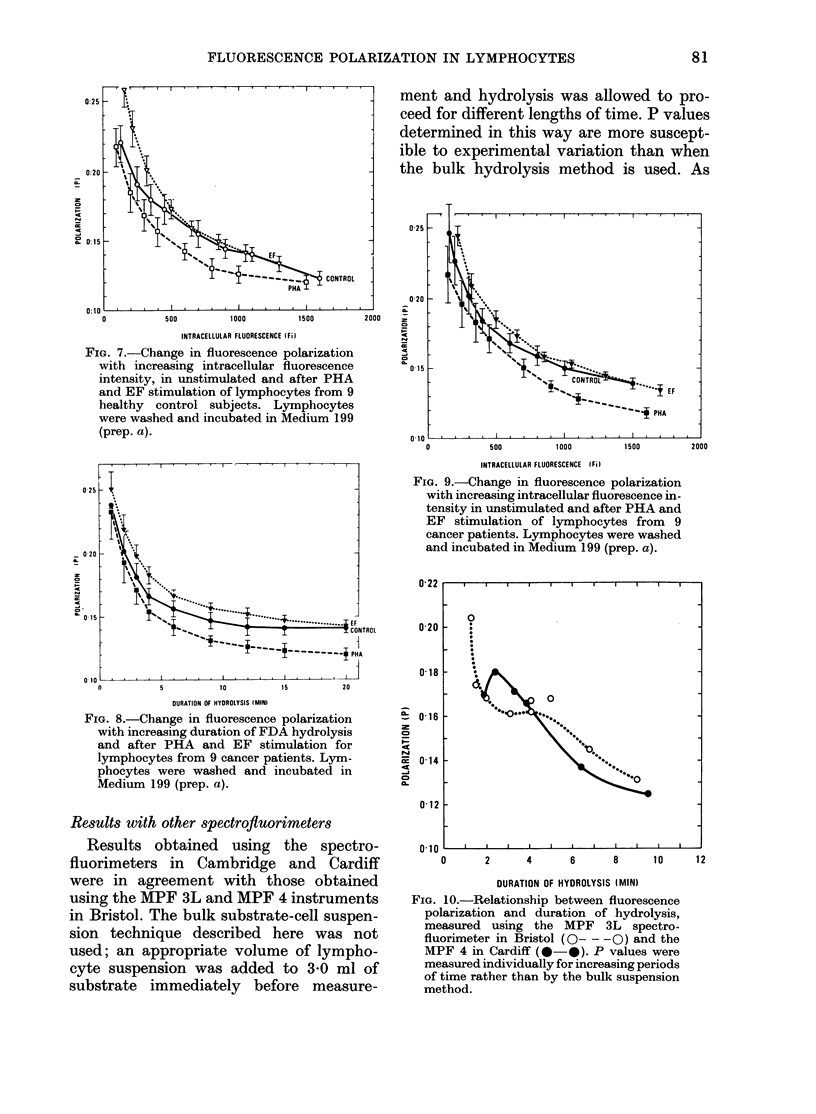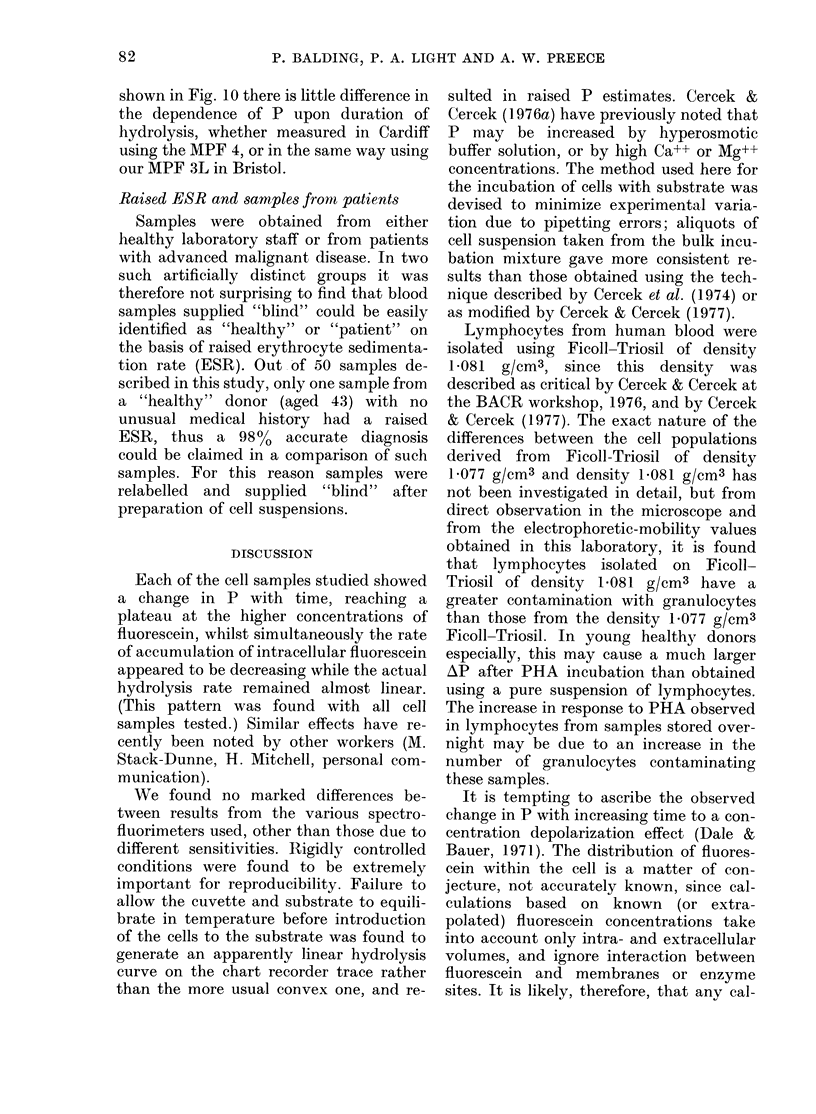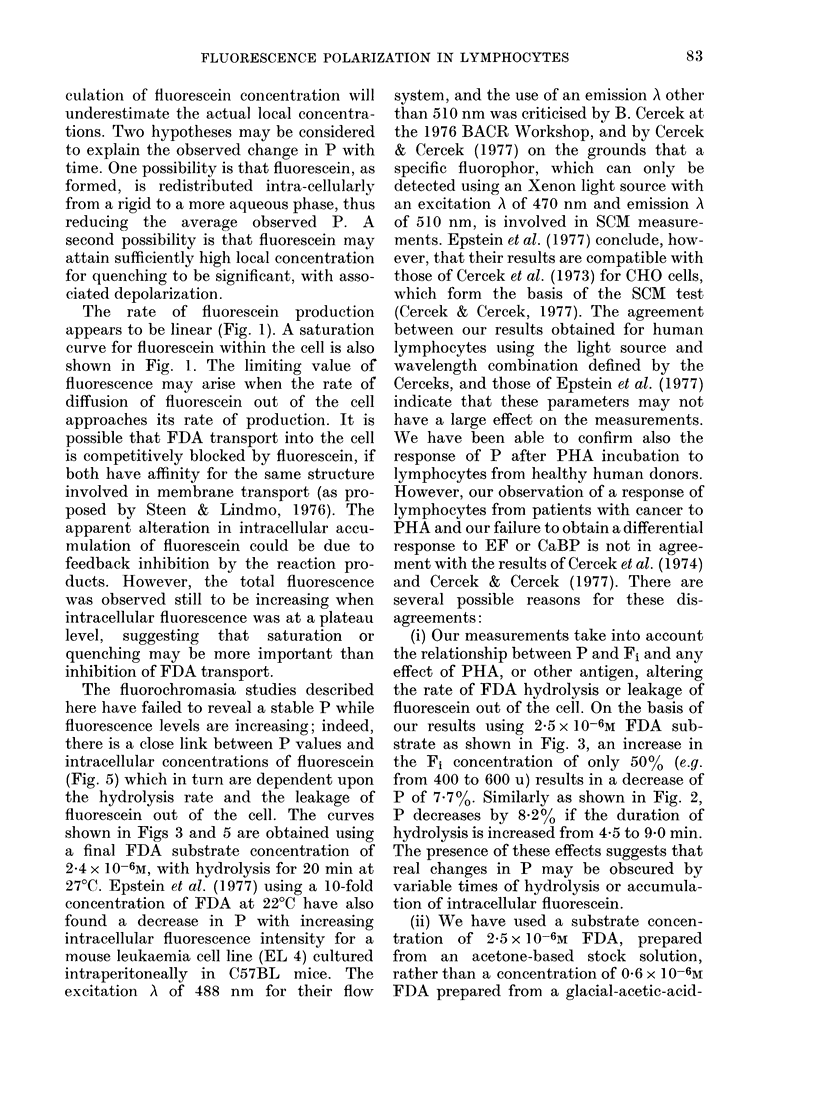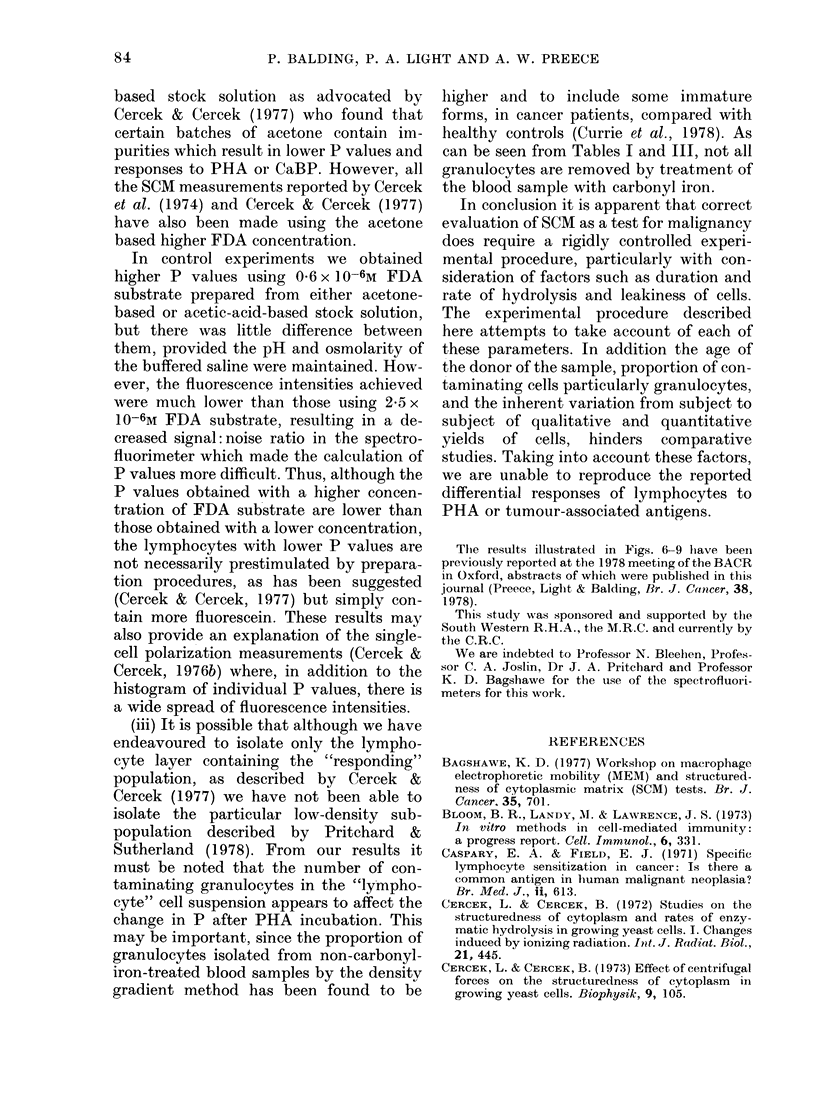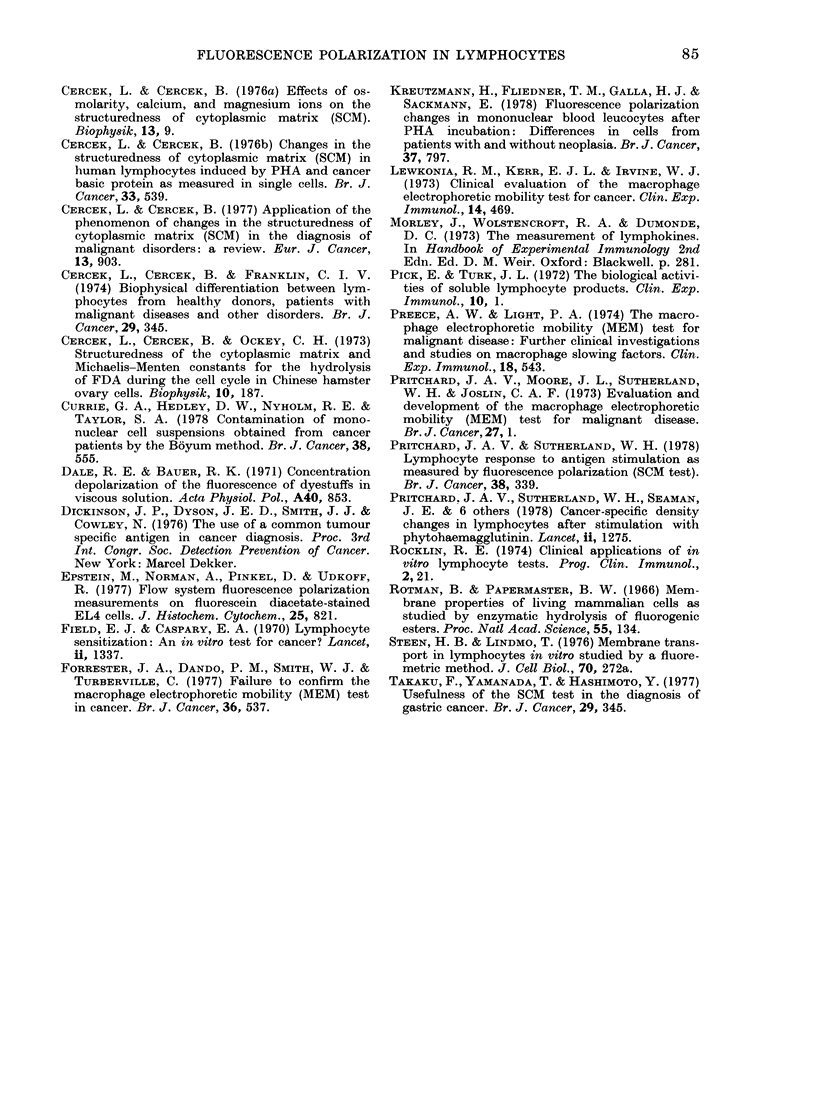# Response of human lymphocytes to PHA and tumour-associated antigens as detected by fluorescence polarization.

**DOI:** 10.1038/bjc.1980.9

**Published:** 1980-01

**Authors:** P. Balding, P. A. Light, A. W. Preece

## Abstract

Fluorescence polarization measurement during the progress of fluorochromasia has been used to study the response of human lymphocytes to phytohaemagglutinin (PHA) and to tumour-associated antigens, as a basis for the detection of malignant disease. Polarization (P) values of both stimulated and unstimulated lymphocytes decreased with increasing intracellular fluorescence intensity, and with the duration of the fluorochromatic reaction. When these effects were taken into account, there was no significant difference in the change of P following stimulation of lymphocytes from 50 cancer patients or healthy subjects; the magnitude of the response was related more to the age of the donor and to the extent of granulocyte contamination of the lymphocyte preparation than to the presence of cancer. There were, however, significant differences in the change in leakage of fluorescein out of the lymphocytes and in the change in hydrolysis rate after PHA stimulation between lymphocytes from healthy individuals and from patients with cancer.


*Br. J. Cancer* (1980) **41**, 73

### RESPONSE OF HUMAN LYMPHOCYTES TO PHA AND TUMOUR-ASSOCIATED ANTIGENS AS DETECTED BY FLUORESCENCE POLARIZATION

P. BALDING, P. A. LIGHT AND A. W. PREECE

*From the Oncology Research Unit, Radiotherapy Centre, Bristol Royal Infirmary, Bristol*

Received 24 April 1979 Accepted 21 September 1979

**Summary.—Fluorescence polarization measurement during the progress of fluorochromasia has been used to study the response of human lymphocytes to phytohaemagglutinin (PHA) and to tumour-associated antigens, as a basis for the detection of malignant disease.**

**Polarization (P) values of both stimulated and unstimulated lymphocytes decreased with increasing intracellular fluorescence intensity, and with the duration of the fluorochromatic reaction. When these effects were taken into account, there was no significant difference in the change of P following stimulation of lymphocytes from 50 cancer patients or healthy subjects; the magnitude of the response was related more to the age of the donor and to the extent of granulocyte contamination of the lymphocyte preparation than to the presence of cancer.**

**There were, however, significant differences in the change in leakage of fluorescein out of the lymphocytes and in the change in hydrolysis rate after PHA stimulation between lymphocytes from healthy individuals and from patients with cancer.**

A NUMBER of *in vitro* methods exist to demonstrate cell-mediated immune responses to antigens in animals and man (Bloom *et al.*, 1973; Rocklin, 1974). The basis of many of these methods is the interaction of sensitized lymphocytes with the specific antigen, to release biologically active susbtances (Pick & Turk, 1972) whose presence may be detected by their effect on other cell types (Morley *et al.*, 1973).

In 1970, Field & Caspary presented evidence that lymphocytes from patients with malignant disease were sensitized in the above manner to basic proteins derived from malignant tumour tissue. They introduced a novel method for detecting lymphocyte sensitization which relied on cell electrophoresis, and subsequently became known as the Macrophage Electrophoretic Mobility (MEM) test (Pritchard *et al.*, 1973). Results with this technique could be obtained more quickly than with any previously described method. The test relied on the *in vitro* reaction of lymphocytes from patients with malignant disease with a cancer basic protein (CaBP) or with a brain-derived protein (encephalitogenic factor, EF) to release a substance which reduced the surface charge on guinea-pig macrophages or other target cells, and could hence be detected by their subsequently reduced electrophoretic mobility. However, although their observations were at least partially confirmed in some laboratories (Pritchard *et al.*, 1973; Preece & Light, 1974), the test was technically difficult, and several groups reported that it was too unreliable to be of any clinical use (Lewkonia *et al.*, 1973; Forrester *et al.*, 1977). The potential applications for a simple rapid test in which malignancy can be detected at an early stage in its development are obvious and far-reaching. Therefore, the description by Cercek *et al.* (1974) of the use of fluorescence-polariza-

Correspondence to Dr P. Balding.

tion measurements to detect lymphocyte sensitization caused widespread interest. In their test system, measurement of fluorescence polarization (P) of fluorescein molecules in the cytoplasmic matrix following fluorochromasia (Rotman & Papermaster, 1966) was used to demonstrate the response of human lymphocytes to the mitogen phytohaemagglutinin (PHA) or to the cancer basic protein (CaBP) and encephalitogenic factor (EF) used in the MEM test. Their earlier studies on yeast cells (Cercek & Cercek, 1972, 1973) and on Chinese hamster ovary cells (Cercek *et al.*, 1973) had indicated that there existed a relationship between fluorescence polarization of fluorescein molecules (produced by enzymatic hydrolysis of the non-fluorescing fluorescein diacetate, FDA) in the cytoplasmic matrix and the average microviscosity of the lymphocyte cytoplasm. Their investigations using this technique were then extended to human lymphocytes, and their results indicated that the responses of lymphocytes from patients with malignant disease to CaBP and EF and to PHA were different from those of lymphocytes from normal subjects. Such responses were observed after a very short exposure to antigen or mitogen; lymphocytes from cancer patients showed a 20% decrease in P value within a 5min exposure to CaBP or EF, and lymphocytes from healthy donors showed a similar response to PHA but not to EF or CaBP. They called this test the Structuredness of Cytoplasmic Matrix (SCM) test.

The SCM test as described by Cercek *et al.* (1974) and subsequently modified (Cercek & Cercek, 1977) has been confirmed by Takaku *et al.* (1977) and Pritchard & Sutherland (1978). The lack of response to PHA of lymphocytes from cancer patients has been confirmed by Kreutzmann *et al.* (1978). In a later study, Pritchard *et al.* (1978) reported that lymphocytes from cancer patients did show a response to PHA, but that the differential between cancer and non-cancer lay in the different responses of high- and low-density lymphocytes. However, Dickinson *et al.* (1976) and Bocklehurst, Pentycross and others (see Bagshawe, 1977) have been unable to confirm the results using the original technique of Cercek *et al.* (1974) and Dickinson, Pentycross and Stack-Dunne (personal communications) have also been unable to reproduce the SCM test using the modified technique of Cercek & Cercek (1977).

This study sought to examine the differential PHA response of lymphocytes from healthy donors and patients with malignant disease, and to attempt to detect any response to CaBP or EF by lymphocytes from such patients, whilst taking note of the progress of fluorochromasia in these cells.

### MATERIALS AND METHODS

### *Preparation of cell suspensions*

Human lymphocytes were prepared from 20 ml peripheral blood containing preservative-free heparin (15 u/ml). Either immediately after collection (preparation technique *a*) or after first allowing the blood to stand overnight at ambient temperature (preparation technique *b*) the blood was incubated with ~100 mg iron carbonyl (Grade SF, GAF Ltd) at 37°C for 20 min with continuous gentle mixing. After sedimentation of the iron carbonyl-containing cells on a magnet, an approximate ESR (erythrocyte sedimentation rate) was noted and the temperature of the blood was adjusted to 25°C (prep. *a*) or 13°C (prep. *b*) by standing in a water bath. The lymphocytes contained in the supernatant were then separated at 25°C (prep. *a*) or 13°C (prep. *b*) on a Ficoll-Triosil solution (density = 1·081 g/cm$^3$, osmolality—320 mOsm and 300 mOsm, respectively, at those temperatures) and centrifuged at 550 *g* for 20 min, as described by Cercek & Cercek (1977). Lymphocytes were collected by removing only the band of cells floating above the Ficoll-Triosil solution with a saline-rinsed Pasteur pipette, care being taken to remove as little plasma or Ficoll-Triosil as possible, and washed twice in sterile saline (Baxter Division, Travenol Labs, Thetford).

Prep. *a* cells were then washed twice in Medium 199 with Earle's salts, 25mM HEPES and L-glutamine (Gibco Biocult Ltd). Prep.

*b* cells were washed twice in PBS (complete Dulbecco phosphate-buffered saline (pH 7·3) from Oxoid Ltd code SR 38 plus code BR 14a) the osmolarity of which was adjusted to 330 mOsm with NaCl, before filtration through a Millipore filter, 0·45$\mu$m pore size (cat. no. HAWP 02500). Lymphocytes were resuspended in fresh Medium 199 or PBS as appropriate, at a concentration of $4 \times 10^6$/ml. Cell suspensions were examined under the phase-contrast microscope, and total lymphocytes, erythrocytes and granulocytes were counted.

At this stage samples were relabelled by a person not involved in the experimental procedure, in order to overcome possible subjective bias associated with the observed raised ESR in blood samples from patients (see later).

*Lymphocyte stimulation*

Cells suspensions were allowed to stand at ambient temperature for about 2 h after preparation. Lymphocytes were then incubated for 30 min at 37°C with (1) 0·1 ml sterile saline for the unstimulated sample, (2) with 0·1 ml of reconstituted reagent grade PHA (Wellcome Reagents) diluted $\times 5$ in sterile saline for PHA stimulation, (3) with 25 $\mu$g of EF or CaBP contained in 0·1 ml sterile saline, for tumour-associated antigen stimulation. EF (from human brain) and CaBP (from carcinoma bronchus tissue obtained *post mortem*) prepared by the method of Caspary & Field (1971) were of known activity, and were used concurrently in the MEM test (Preece & Light, 1974) during the course of this investigation.

*Measurement of fluorescence polarization and intensity*

Fluorescein diacetate (FDA; Koch-Light Laboratories) was dissolved in acetone ("Aristar" Grade; British Drug Houses) at a concentration of 5 mg/ml. For use as a substrate in the spectrofluorimeter, 10 $\mu$l of this solution was added to 50 ml of Millipore-filtered PBS (osmolarity not adjusted to 330 m Osm for prep. *a* cells only); the substrate solution containing $2{\cdot}4 \times 10^{-6}$M of FDA was equilibrated to 27°C before the addition of cells to an appropriate volume of substrate to give a final lymphocyte concentration of $1{\cdot}3 \times 10^5$/ml. This cell suspension was maintained at 27°C and 3ml aliquots were pipetted into the cuvette, also maintained at 27°C, after a known duration of hydrolysis. Subsequent filtration of the suspension was carried out using 0·22$\mu$m pore-size Millipore filters (Cat. No. GSWP 02500) in Millipore Swinnex 25 holders, or 0·45$\mu$m pore-size filters (Cat. No. HAWP 01300) in Swinnex 15 holders, using suction limited to 50 kPa.

Measurements of fluorescence polarization were made with two different instruments: Perkin Elmer MPF 3L Spectrofluorimeter, and later, for part of the series using cells of prep *b*, a Perkin Elmer MPF 4. The excitation wavelength used was 470 nm (20 nm bandwidth) and the emission wavelength used was 510 nm (10 nm bandwidth). A Baird-Atomic FITC barrier filter (cut off below 505 nm) was used in the emission light path to reduce unwanted scattered light to negligible levels (less than 2% at the maximum spectrofluorimeter gain setting used). Excitation intensity was increased in both instruments by incorporating a $\frac{1}{2}$-wave plate (Polaroid, Polarizers, U.K.) at the correct angle in the excitation beam; a $\frac{1}{4}$-wave plate (Polaroid) was also used in the emission beam of the MPF 3L to correct the G factor from 0·77 to 1·0, to facilitate interpretation of the chart recorder traces. Polaroid HN 32 polarising filters (cross-over extinction less than 0·02% of the parallel transmission) were used as analysing filters.

For comparison, some studies were carried out with a Perkin Elmer 4F spectrofluorimeter at Cambridge (by kind permission of Prof. N. Blechen) using the same experimental procedures as described above, and further studies were carried out with another Perkin Elmer MPF 4 at Cardiff, (by kind permission of Dr J. A. V. Pritchard) but using procedures as described by Cercek *et al.* (1974) as modified by Cercek & Cercek (1977); FDA was used at a concentration of $2{\cdot}4 \times 10^{-6}$ M. In all instruments a cuvette with a light path of 10 mm was used.

Polarization of emitted light, P, was measured at right angles to the polarized excitation beam as:

$$P = \frac{I_{VV} - GI_{VH}}{I_{VV} + GI_{VH}}$$

where $I_{VV}$ and $I_{VH}$ are the fluorescent intensities observed parallel and perpendicular respectively to the polarization of the excitation beam, and G is the grating factor determined experimentally for each machine. Total fluorescence intensity $F_t$ and extracellular fluorescence intensity $F_s$ were calculated from the basic equation:

$$F = I_{VV} + 2\ GI_{VH} - F_0$$

where $F_0$ is the contribution to the fluorescence intensity from spontaneous hydrolysis of the FDA substrate, measured after an appropriate time using substrate solution without added cell suspension. The intracellular fluorescence intensity, $F_i = F_t - F_s$. When $F_i = 1000$ u, the equivalent average concentration of fluorescein per cell is $\sim 2{\cdot}5 \times 10^{-4}$M (1000 u of fluorescence is emitted by $8 \times 10^{-9}$M NaF). Change in polarization values ($\Delta$P) with duration of hydrolysis and intracellular fluorescence intensity was calculated as follows: The P for at least 8 increasing lengths of time of hydrolysis was plotted against duration of hydrolysis and against the intracellular fluorescence value ($F_i$) for each value of P. The P values corresponding to 2 min, 5 min, 10 min, 15 min, and 20 min hydrolysis were taken from the graph for the unstimulated cells and compared with values obtained for equal times using "stimulated" cells. The mean difference was expressed as %$\Delta$P (time). Similarly %$\Delta$P ($F_i$) was determined from $F_i$ values of 200, 600 and 1200 u of fluorescence. The % change in leakage with time and with $F_i$ was calculated in the same way. The % change in total hydrolysis rate (%$\Delta F_t$) was calculated by comparing the average rate/min over 20 min of hydrolysis; the %$\Delta$P with time for unstimulated cells was calculated by expressing the difference between the P at 20 min and the P at 1 min as a % of the latter.

*Subjects*

The malignant group consisted of inpatients with carcinomas, sarcomas and lymphoma. Each had diagnosed disease with tumour present, had not yet started radiotherapy nor had had recent or extensive chemotherapy; age range was 27–84 years. The healthy subjects were volunteer hospital staff selected to cover a wide age range (25–55 years).

### RESULTS

### *Fluorochromasia in cell suspensions*

Addition of lymphocytes to FDA substrate produced an immediate increase in fluorescent intensity; the minimum duration of hydrolysis which permitted calculation of fluorescence and P was found to be about 1 min. As shown for a typical healthy control sample in Fig. 1, the rate

FIG. 1.—Fluorochromasia in human lymphocytes and the relationship between polarization of fluorescence and duration of FDA hydrolysis at 27°C.

of hydrolysis of FDA to fluorescein is almost linear, but the rate of accumulation of intracellular fluorescein ($F_i$) gradually decreased to a plateau after 14 min. The extracellular ("supernatant") fluorescence gradually increased from 12% to 56% of total fluorescence ($F_t$) over 20 min. P tends towards a plateau simultaneously with the $F_i$ values. Reproducibility for unstimulated lymphocytes from a single

FIG. 2.—Relationship between polarization of fluorescence and duration of hydrolysis for unstimulated lymphocytes from 16 healthy controls (○—○). Results for lymphocytes from 16 patients with malignant disease (●) are included for comparison. (± s.e.).

FIG. 3.—Relationship between polarization of fluorescence and intracellular fluorescence for unstimulated lymphocytes from 16 healthy controls (○—○). Results for lymphocytes from 16 patients with malignant disease (●) are included for comparison. (±s.e.).

donor tested 3 times on the same day was better than ±2·5% (s.d.). The mean variation in P for lymphocytes of 16 healthy controls corresponding to increased duration and $F_i$ values is shown in Figs 2 and 3. P values of unstimulated lymphocytes from 16 patients with malignant diseases are included for comparison.

Thus the experimental technique used here permitted the calculation of a number of different parameters in addition to P and %ΔP after incubation of lymphocytes with PHA, CaBP or EF.

Such parameters included:

(i) % change in average hydrolysis rate over 20 min ($\Delta F_t$/min) for stimulated compared with unstimulated lymphocytes.

(ii) % change in P of unstimulated lymphocytes with duration of hydrolysis. (ΔP 1–20.)

(iii) % change in P of stimulated compared with unstimulated lymphocytes averaged over several values for duration of hydrolysis (ΔP (time)) and (iv) averaged over several values for intracellular fluorescence intensity (ΔP ($F_i$)).

(v) Δ leakiness (time) and (vi) Δ leakiness ($F_i$): % change in leakiness of stimulated lymphocytes calculated in the same way as (iii) and (iv).

*Effect of overnight storage of blood samples on P*

Initially lymphocytes were prepared from blood samples immediately after collection. However, we learned that lymphocytes used in the SCM test were often isolated from blood samples collected from donors on the previous day (L. Cercek, personal communication). Therefore blood samples were intentionally stored overnight at ambient temperature prior to isolation of lymphocytes. As shown in Fig. 4, such treatment produced an increased response to PHA, so all samples were stored overnight prior to isolation of lymphocytes using prep. *b*.

FIG. 4.—Effect of overnight storage of blood samples on change in fluorescence polarization following PHA stimulation of lymphocytes from healthy controls (mean of 2 experiments on 2 donors ±s.e.).

*Effect of incubation with PHA*

Results for healthy controls and for 16 patients with malignant disease are shown in Tables I to IV.

The mean percentage change in P with duration of hydrolysis after incubation with PHA for 30 min at 37°C was a decrease of 8·4 ± 1·8 (s.e.) for patient samples, compared to a decrease of 11·9 ± 1·5 for healthy control samples. This difference is not significant ($0{\cdot}05 < P < 0{\cdot}1$). However, there is a simultaneous change in FDA hydrolysis rate (+10·3 ± 4·4 for healthy control samples, compared with +0·6 ± 3·3 in patient samples) and in

TABLE I.—*Cell yields and % change in polarization of fluorescence with time of unstimulated lymphocytes and after incubation with PHA or EF. Samples from healthy individuals*

| Age and sex | ΔP (1–20 min) unstim. | ΔP (time) PHA | ΔP (time) EF | ΔP ($F_i$) PHA | ΔP ($F_i$) EF | Cell yield $\times 10^{-6}$ | % cells* Ly | % cells* E | % cells* G |
|---|---|---|---|---|---|---|---|---|---|
| 25F | −23·5 | − 7·1 | — | − 2·1 | — | 20 | 80 | 15 | 5 |
| 26F | −20·5 | − 4·9 | + 2·1 | + 3·8 | +10·7 | 24 | — | — | — |
| 29M | −18·9 | −15·6 | + 4·0 | −11·6 | +13·5 | 24 | — | — | — |
| 30M | −11·4 | −16·3 | — | −12·4 | — | 18 | — | — | — |
| 33M | −19·5 | −13·5 | — | −11·2 | — | 32 | — | — | — |
| 34M | −29·6 | −11·6 | + 2·0 | − 4·9 | + 5·8 | 22 | — | — | — |
| 36M | −22·6 | −20·1 | — | − 9·8 | — | 24 | — | — | — |
| 38M | −16·4 | −15·1 | — | −10·9 | — | 22 | — | — | — |
| 43F | −32·2 | −11·6 | — | −13·0 | — | 21 | 79 | 15 | 6 |
| 47M | −26·9 | − 9·4 | − 1·1 | − 0·5 | − 2·5 | 22 | 79 | 16 | 5 |
| 50M | −26·4 | + 3·1 | — | +11·7 | — | 16 | 75 | 20 | 5 |
| 51F | −15·6 | −19·5 | — | − 6·5 | — | 16 | 50 | 24 | 26 |
| 51M | −21·8 | −15·3 | − 9·3 | −14·0 | − 7·0 | 20 | 76 | 12 | 12 |
| 54M | −22·8 | −19·4 | — | −16·5 | — | 10 | 65 | 25 | 10 |
| 54M | −23·7 | − 4·5 | + 2·7 | + 4·2 | + 5·8 | 19 | 74 | 18 | 8 |
| 55F | −27·6 | − 9·7 | — | −12·1 | — | 24 | 78 | 17 | 5 |
| Mean | −22·5 | −11·9 | + 0·1 | − 6·6 | + 4·4 | 21 | | | |
| s.e. | 1·3 | 1·5 | 1·8 | 1·9 | 2·9 | 1·2 | | | |

* Ly = Lymphocytes; E = Erythrocytes; G = granulocytes.

TABLE II.—*Change in FDA hydrolysis rate and in leakage of fluorescein out of lymphocytes after incubation with PHA or EF, expressed as % of unstimulated values. Samples from healthy individuals*

| Age and sex | Δ$F_t$ rate/min PHA | Δ$F_t$ rate/min EF | Δ Leak (time) PHA | Δ Leak (time) EF | Δ Leak ($F_i$) PHA | Δ Leak ($F_i$) EF |
|---|---|---|---|---|---|---|
| 25F | −7·5 | — | −8·8 | — | −7·3 | — |
| 26F | +13·5 | −7·8 | −4·4 | +1·0 | −6·3 | +2·3 |
| 29M | +12·2 | +5·0 | −5·8 | +2·2 | −11·0 | 0 |
| 30M | +5·6 | — | −3·6 | — | −3·7 | — |
| 33M | +4·0 | — | −10·6 | — | −9·3 | — |
| 34M | +5·2 | −0·6 | −6·2 | −0·8 | −13·7 | −3·7 |
| 36M | +12·6 | — | −7·2 | — | −9·5 | — |
| 38M | +4·4 | — | −7·4 | — | −7·3 | — |
| 43F | −2·0 | — | −6·6 | — | −8·7 | — |
| 47M | −6·1 | +2·8 | −0·9 | +4·1 | −1·6 | +0·3 |
| 50M | +36·6 | — | +1·2 | — | −3·7 | — |
| 51F | +18·8 | — | −4·0 | — | −3·7 | — |
| 51M | −0·8 | +15·0 | −1·2 | +0·4 | +1·3 | +1·7 |
| 54M | +64·0 | — | −4·8 | — | −9·7 | — |
| 54M | +12·1 | +11·3 | −3·8 | +5·6 | −7·0 | +1·7 |
| 55F | −8·6 | — | −6·4 | — | −2·0 | — |
| Mean | +10·3 | +4·9 | −5·1 | +2·1 | −6·5 | +0·4 |
| s.e. | 4·4 | 3·1 | 0·7 | 0·9 | 1·0 | 0·8 |

leakage of the fluorescein out of the lymphocytes ($-5{\cdot}1 \pm 0{\cdot}7$ with duration and $-6{\cdot}5 \pm 1{\cdot}0$ with $F_i$ levels for healthy control samples, and zero change with duration or $+0{\cdot}8 \pm 1{\cdot}3$ with $F_i$ levels for patient samples) which taken together give an increase in $F_i$, especially in healthy control samples. As shown in Fig. 5 the change in P is proportional to the $F_i$ level in unstimulated lymphocytes, so a comparison of P changes has to be made on the basis of similar $F_i$ intensities. Such values of %ΔP for healthy control samples and patient samples respectively are $-6{\cdot}6 \pm 1{\cdot}9$ and $-7{\cdot}7 \pm 1{\cdot}9$.

Thus although there is a real decrease

TABLE III.—*Cell yields and % change in polarization of fluorescence (ΔP) of unstimulated lymphocytes and after incubation with PHA or EF. Samples from patients with malignant disease*

| Age and sex | ΔP (1–20 min) unstim. | ΔP (time) PHA | ΔP (time) EF | ΔP ($F_i$) PHA | ΔP ($F_i$) EF | Cell $\times 10^{-6}$ yield | % cells Ly | % cells E | % cells G |
|---|---|---|---|---|---|---|---|---|---|
| 27F | −23·0 | −13·2 | — | −15·2 | — | 7 | 59 | 26 | 15 |
| 30M | −25·7 | −5·6 | −2·7 | −1·6 | −2·6 | 15 | 72 | 19 | 9 |
| 33M | −25·3 | −4·7 | +5·6 | −3·3 | +12·2 | 10 | 59 | 35 | 6 |
| 43M | −19·3 | +2·4 | +6·8 | +7·4 | +9·4 | 17 | 78 | 11 | 11 |
| 47F | −20·6 | −5·7 | — | −6·2 | — | 159* | 89 | 6 | 5 |
| 52F | −24·3 | −15·2 | — | −19·4 | — | 17 | 48 | 49 | 3 |
| 55F | −15·9 | −11·7 | — | −10·2 | — | 10 | — | — | 12 |
| 61M | −24·9 | −6·3 | +1·8 | −6·8 | +1·0 | 12 | — | — | 7 |
| 65M | −18·5 | −11·4 | — | −12·3 | — | 24 | 79 | 15 | 6 |
| 67M | −34·1 | +0·6 | — | +2·4 | — | 15 | — | — | 5 |
| 69M | −14·9 | −5·9 | — | −0·9 | — | 11 | — | — | 10 |
| 70M | −16·7 | −8·2 | +0·8 | −4·3 | +0·2 | 18 | 57 | 22 | 21 |
| 71M | −17·8 | −6·3 | — | −2·5 | — | 14 | 29 | 67 | 4 |
| 73F | −29·1 | −27·0 | — | −21·0 | — | 14 | — | — | 25 |
| 81M | −16·2 | −5·0 | — | −13·1 | — | 17 | — | — | 3 |
| 84F | −32·2 | −15·7 | — | −15·6 | — | 17 | 65 | 25 | 12 |
| Mean | −22·4 | −8·4 | +2·5 | −7·7 | +4·0 | 14·5 | | | |
| s.e. | 1·4 | 1·8 | 1·5 | 1·9 | 2·6 | 1·0 | | | |

* Not included in mean of cell yield—patient had leukaemia.

TABLE IV.—*Change in FDA hydrolysis rate and in leakage of fluorescein out of lymphocytes after incubation with PHA or EF, expressed as % of unstimulated values. Samples from patients with malignant disease*

| Age and sex | $\Delta F_t$ rate/min PHA | $\Delta F_t$ rate/min EF | Δ Leak (time) PHA | Δ Leak (time) EF | Δ Leak ($F_i$) PHA | Δ Leak ($F_i$) EF |
|---|---|---|---|---|---|---|
| 27F | −7·0 | — | −3·8 | — | −1·3 | — |
| 30M | +7·0 | −5·0 | −6·0 | +0·2 | −5·0 | +2·3 |
| 33M | +11·0 | +3·0 | +1·6 | +0·2 | +1·3 | +1·6 |
| 43M | +17·0 | +5·0 | −2·2 | −0·2 | −2·7 | +1·0 |
| 47F | −0·8 | — | +3·6 | — | +4·0 | — |
| 52F | +2·0 | — | −2·6 | — | −11·3 | — |
| 55F | −26·0 | — | −3·8 | — | −4·3 | — |
| 61M | +24·0 | −9·0 | −10·2 | −2·6 | −1·0 | −1·3 |
| 65M | −8·0 | — | +3·0 | — | +1·3 | — |
| 67M | +2·0 | — | 0·0 | — | +1·0 | — |
| 69F | +13·0 | — | −4·6 | — | −5·0 | — |
| 70M | −16·0 | −15·0 | +3·0 | +11·2 | −1·0 | +15·3 |
| 71M | +17·0 | — | +3·6 | — | +3·0 | — |
| 73F | +10·0 | — | +5·2 | — | +1·3 | — |
| 81M | −15·0 | — | +2·2 | — | +12·3 | — |
| 84F | −3·0 | — | +0·6 | — | −1·3 | — |
| Mean | +0·6 | −4·2 | 0·0 | +1·8 | −0·8 | +3·8 |
| s.e. | 3·3 | 1·9 | 0·8 | 2·2 | 1·2 | 2·6 |

in P corresponding to a net decrease in cytoplasmic viscosity of the lymphocyte, this change is relatively small, and is almost identical in samples from both healthy controls and cancer patients. From these results it also appears that there is a general trend to an increase in the magnitude of ΔP after PHA incubation, both with decreasing age of the sample donor, and with an increase in granulocyte contamination (a plot of %ΔP *vs* % granulocyte contamination has a correlation of 0·61 and a slope of 0·64). Erythrocyte contamination does not appear to influence ΔP, but the PHA response might be reduced when much larger numbers of erythrocytes are present, since the concentration of PHA available

FIG. 5.—Dependence of polarization changes on intracellular fluorescence levels for unstimulated lymphocytes from 16 healthy donors.

to interact with lymphocytes would be reduced by absorption. For the samples shown in Tables I and III, the effect of erythrocyte contamination is insignificant compared to that caused by granulocyte contamination.

### *Effect of incubation with CaBP and EF*

The responses to both CaBP and EF were identical, so only results with EF are presented. There was very little difference in response of lymphocytes isolated from either patients or controls. As found using PHA any small difference disappeared when ΔP with $F_i$ was compared. Similarly, the effects of incubation with EF on FDA hydrolysis rate and leakage of fluorescein out of the cells were significantly different, but it is unlikely that these differences could be of any diagnostic use.

The mean values for % leakage of fluorescein out of unstimulated lymphocytes from both healthy controls and patients with cancer were not significantly different ($37{\cdot}0 \pm 1{\cdot}0$ and $36{\cdot}0 \pm 1{\cdot}6$ respectively) nor was the ΔP with duration of hydrolysis ($-22{\cdot}5 \pm 1{\cdot}3$ and $-22{\cdot}4 \pm 1{\cdot}4$ respectively).

### *Effect of variation in preparation of lymphocytes*

For the purpose of comparing the earlier (prep. *a*) and later (prep. *b*) techniques for cell preparation, samples from 9 healthy controls and 9 patients with cancer were processed using the original technique for cell preparation (prep. *a*) described by Cercek *et al.* (1974) but with two modifications. (i) We used Ficoll–Triosil of density 1·081 g/cm³ originally used in error by the group but subsequently shown to be critical (reported by L. Cercek at the BACR workshop in 1976; see Bagshawe, 1977). (ii) We used Gibco Biocult Medium 199 containing 2mM HEPES and L-glutamine rather than TC 199 (Wellcome Reagents) since we found the pH of the latter to be unstable during incubation of lymphocytes at 37°C and the cells were prone to clumping. Results showing P against duration of hydrolysis and $F_i$ for unstimulated lymphocytes, and after incubation with PHA or EF are shown in Figs 6–9. The results were not significantly different comparing healthy controls with patients, nor were they different when comparing the cell preparation techniques (*a* and *b*) except that when the higher-osmolarity PBS was used for cell suspension, there was a smaller increase in P after incubation with EF.

FIG. 6.—Change in fluorescence polarization with increasing duration of FDA hydrolysis and after PHA and EF stimulation for lymphocytes from 9 healthy control subjects. Lymphocytes were washed and incubated in Medium 199 (prep. *a*).

FIG. 7.—Change in fluorescence polarization with increasing intracellular fluorescence intensity, in unstimulated and after PHA and EF stimulation of lymphocytes from 9 healthy control subjects. Lymphocytes were washed and incubated in Medium 199 (prep. *a*).

FIG. 8.—Change in fluorescence polarization with increasing duration of FDA hydrolysis and after PHA and EF stimulation for lymphocytes from 9 cancer patients. Lymphocytes were washed and incubated in Medium 199 (prep. *a*).

### *Results with other spectrofluorimeters*

Results obtained using the spectrofluorimeters in Cambridge and Cardiff were in agreement with those obtained using the MPF 3L and MPF 4 instruments in Bristol. The bulk substrate-cell suspension technique described here was not used; an appropriate volume of lymphocyte suspension was added to 3·0 ml of substrate immediately before measurement and hydrolysis was allowed to proceed for different lengths of time. P values determined in this way are more susceptible to experimental variation than when the bulk hydrolysis method is used. As

FIG. 9.—Change in fluorescence polarization with increasing intracellular fluorescence intensity in unstimulated and after PHA and EF stimulation of lymphocytes from 9 cancer patients. Lymphocytes were washed and incubated in Medium 199 (prep. *a*).

FIG. 10.—Relationship between fluorescence polarization and duration of hydrolysis, measured using the MPF 3L spectrofluorimeter in Bristol (○– – –○) and the MPF 4 in Cardiff (●—●). *P* values were measured individually for increasing periods of time rather than by the bulk suspension method.

shown in Fig. 10 there is little difference in the dependence of P upon duration of hydrolysis, whether measured in Cardiff using the MPF 4, or in the same way using our MPF 3L in Bristol.

*Raised ESR and samples from patients*

Samples were obtained from either healthy laboratory staff or from patients with advanced malignant disease. In two such artificially distinct groups it was therefore not surprising to find that blood samples supplied "blind" could be easily identified as "healthy" or "patient" on the basis of raised erythrocyte sedimentation rate (ESR). Out of 50 samples described in this study, only one sample from a "healthy" donor (aged 43) with no unusual medical history had a raised ESR, thus a 98% accurate diagnosis could be claimed in a comparison of such samples. For this reason samples were relabelled and supplied "blind" after preparation of cell suspensions.

### DISCUSSION

Each of the cell samples studied showed a change in P with time, reaching a plateau at the higher concentrations of fluorescein, whilst simultaneously the rate of accumulation of intracellular fluorescein appeared to be decreasing while the actual hydrolysis rate remained almost linear. (This pattern was found with all cell samples tested.) Similar effects have recently been noted by other workers (M. Stack-Dunne, H. Mitchell, personal communication).

We found no marked differences between results from the various spectrofluorimeters used, other than those due to different sensitivities. Rigidly controlled conditions were found to be extremely important for reproducibility. Failure to allow the cuvette and substrate to equilibrate in temperature before introduction of the cells to the substrate was found to generate an apparently linear hydrolysis curve on the chart recorder trace rather than the more usual convex one, and resulted in raised P estimates. Cercek & Cercek (1976*a*) have previously noted that P may be increased by hyperosmotic buffer solution, or by high $Ca^{++}$ or $Mg^{++}$ concentrations. The method used here for the incubation of cells with substrate was devised to minimize experimental variation due to pipetting errors; aliquots of cell suspension taken from the bulk incubation mixture gave more consistent results than those obtained using the technique described by Cercek *et al.* (1974) or as modified by Cercek & Cercek (1977).

Lymphocytes from human blood were isolated using Ficoll–Triosil of density 1·081 g/cm$^3$, since this density was described as critical by Cercek & Cercek at the BACR workshop, 1976, and by Cercek & Cercek (1977). The exact nature of the differences between the cell populations derived from Ficoll-Triosil of density 1·077 g/cm$^3$ and density 1·081 g/cm$^3$ has not been investigated in detail, but from direct observation in the microscope and from the electrophoretic-mobility values obtained in this laboratory, it is found that lymphocytes isolated on Ficoll–Triosil of density 1·081 g/cm$^3$ have a greater contamination with granulocytes than those from the density 1·077 g/cm$^3$ Ficoll–Triosil. In young healthy donors especially, this may cause a much larger $\Delta P$ after PHA incubation than obtained using a pure suspension of lymphocytes. The increase in response to PHA observed in lymphocytes from samples stored overnight may be due to an increase in the number of granulocytes contaminating these samples.

It is tempting to ascribe the observed change in P with increasing time to a concentration depolarization effect (Dale & Bauer, 1971). The distribution of fluorescein within the cell is a matter of conjecture, not accurately known, since calculations based on known (or extrapolated) fluorescein concentrations take into account only intra- and extracellular volumes, and ignore interaction between fluorescein and membranes or enzyme sites. It is likely, therefore, that any calculation of fluorescein concentration will underestimate the actual local concentrations. Two hypotheses may be considered to explain the observed change in P with time. One possibility is that fluorescein, as formed, is redistributed intra-cellularly from a rigid to a more aqueous phase, thus reducing the average observed P. A second possibility is that fluorescein may attain sufficiently high local concentration for quenching to be significant, with associated depolarization.

The rate of fluorescein production appears to be linear (Fig. 1). A saturation curve for fluorescein within the cell is also shown in Fig. 1. The limiting value of fluorescence may arise when the rate of diffusion of fluorescein out of the cell approaches its rate of production. It is possible that FDA transport into the cell is competitively blocked by fluorescein, if both have affinity for the same structure involved in membrane transport (as proposed by Steen & Lindmo, 1976). The apparent alteration in intracellular accumulation of fluorescein could be due to feedback inhibition by the reaction products. However, the total fluorescence was observed still to be increasing when intracellular fluorescence was at a plateau level, suggesting that saturation or quenching may be more important than inhibition of FDA transport.

The fluorochromasia studies described here have failed to reveal a stable P while fluorescence levels are increasing; indeed, there is a close link between P values and intracellular concentrations of fluorescein (Fig. 5) which in turn are dependent upon the hydrolysis rate and the leakage of fluorescein out of the cell. The curves shown in Figs 3 and 5 are obtained using a final FDA substrate concentration of $2{\cdot}4 \times 10^{-6}$M, with hydrolysis for 20 min at 27°C. Epstein *et al.* (1977) using a 10-fold concentration of FDA at 22°C have also found a decrease in P with increasing intracellular fluorescence intensity for a mouse leukaemia cell line (EL 4) cultured intraperitoneally in C57BL mice. The excitation $\lambda$ of 488 nm for their flow system, and the use of an emission $\lambda$ other than 510 nm was criticised by B. Cercek at the 1976 BACR Workshop, and by Cercek & Cercek (1977) on the grounds that a specific fluorophor, which can only be detected using an Xenon light source with an excitation $\lambda$ of 470 nm and emission $\lambda$ of 510 nm, is involved in SCM measurements. Epstein *et al.* (1977) conclude, however, that their results are compatible with those of Cercek *et al.* (1973) for CHO cells, which form the basis of the SCM test (Cercek & Cercek, 1977). The agreement between our results obtained for human lymphocytes using the light source and wavelength combination defined by the Cerceks, and those of Epstein *et al.* (1977) indicate that these parameters may not have a large effect on the measurements. We have been able to confirm also the response of P after PHA incubation to lymphocytes from healthy human donors. However, our observation of a response of lymphocytes from patients with cancer to PHA and our failure to obtain a differential response to EF or CaBP is not in agreement with the results of Cercek *et al.* (1974) and Cercek & Cercek (1977). There are several possible reasons for these disagreements:

(i) Our measurements take into account the relationship between P and $F_i$ and any effect of PHA, or other antigen, altering the rate of FDA hydrolysis or leakage of fluorescein out of the cell. On the basis of our results using $2{\cdot}5 \times 10^{-6}$M FDA substrate as shown in Fig. 3, an increase in the $F_i$ concentration of only 50% (*e.g.* from 400 to 600 u) results in a decrease of P of 7·7%. Similarly as shown in Fig. 2, P decreases by 8·2% if the duration of hydrolysis is increased from 4·5 to 9·0 min. The presence of these effects suggests that real changes in P may be obscured by variable times of hydrolysis or accumulation of intracellular fluorescein.

(ii) We have used a substrate concentration of $2{\cdot}5 \times 10^{-6}$M FDA, prepared from an acetone-based stock solution, rather than a concentration of $0{\cdot}6 \times 10^{-6}$M FDA prepared from a glacial-acetic-acidbased stock solution as advocated by Cercek & Cercek (1977) who found that certain batches of acetone contain impurities which result in lower P values and responses to PHA or CaBP. However, all the SCM measurements reported by Cercek *et al.* (1974) and Cercek & Cercek (1977) have also been made using the acetone based higher FDA concentration.

In control experiments we obtained higher P values using $0{\cdot}6 \times 10^{-6}$M FDA substrate prepared from either acetone-based or acetic-acid-based stock solution, but there was little difference between them, provided the pH and osmolarity of the buffered saline were maintained. However, the fluorescence intensities achieved were much lower than those using $2{\cdot}5 \times 10^{-6}$M FDA substrate, resulting in a decreased signal:noise ratio in the spectrofluorimeter which made the calculation of P values more difficult. Thus, although the P values obtained with a higher concentration of FDA substrate are lower than those obtained with a lower concentration, the lymphocytes with lower P values are not necessarily prestimulated by preparation procedures, as has been suggested (Cercek & Cercek, 1977) but simply contain more fluorescein. These results may also provide an explanation of the single-cell polarization measurements (Cercek & Cercek, 1976*b*) where, in addition to the histogram of individual P values, there is a wide spread of fluorescence intensities.

(iii) It is possible that although we have endeavoured to isolate only the lymphocyte layer containing the "responding" population, as described by Cercek & Cercek (1977) we have not been able to isolate the particular low-density subpopulation described by Pritchard & Sutherland (1978). From our results it must be noted that the number of contaminating granulocytes in the "lymphocyte" cell suspension appears to affect the change in P after PHA incubation. This may be important, since the proportion of granulocytes isolated from non-carbonyl-iron-treated blood samples by the density gradient method has been found to be higher and to include some immature forms, in cancer patients, compared with healthy controls (Currie *et al.*, 1978). As can be seen from Tables I and III, not all granulocytes are removed by treatment of the blood sample with carbonyl iron.

In conclusion it is apparent that correct evaluation of SCM as a test for malignancy does require a rigidly controlled experimental procedure, particularly with consideration of factors such as duration and rate of hydrolysis and leakiness of cells. The experimental procedure described here attempts to take account of each of these parameters. In addition the age of the donor of the sample, proportion of contaminating cells particularly granulocytes, and the inherent variation from subject to subject of qualitative and quantitative yields of cells, hinders comparative studies. Taking into account these factors, we are unable to reproduce the reported differential responses of lymphocytes to PHA or tumour-associated antigens.

The results illustrated in Figs. 6–9 have been previously reported at the 1978 meeting of the BACR in Oxford, abstracts of which were published in this journal (Preece, Light & Balding, *Br. J. Cancer*, **38**, 1978).

This study was sponsored and supported by the South Western R.H.A., the M.R.C. and currently by the C.R.C.

We are indebted to Professor N. Bleehen, Professor C. A. Joslin, Dr J. A. Pritchard and Professor K. D. Bagshawe for the use of the spectrofluorimeters for this work.

### REFERENCES

Bagshawe, K. D. (1977) Workshop on macrophage electrophoretic mobility (MEM) and structuredness of cytoplasmic matrix (SCM) tests. *Br. J. Cancer*, **35**, 701.

Bloom, B. R., Landy, M. & Lawrence, J. S. (1973) *In vitro* methods in cell-mediated immunity: a progress report. *Cell. Immunol.*, **6**, 331.

Caspary, E. A. & Field, E. J. (1971) Specific lymphocyte sensitization in cancer: Is there a common antigen in human malignant neoplasia? *Br. Med. J.*, **ii**, 613.

Cercek, L. & Cercek, B. (1972) Studies on the structuredness of cytoplasm and rates of enzymatic hydrolysis in growing yeast cells. I. Changes induced by ionizing radiation. *Int. J. Radiat. Biol.*, **21**, 445.

Cercek, L. & Cercek, B. (1973) Effect of centrifugal forces on the structuredness of cytoplasm in growing yeast cells. *Biophysik*, **9**, 105.

CERCEK, L. & CERCEK, B. (1976*a*) Effects of osmolarity, calcium, and magnesium ions on the structuredness of cytoplasmic matrix (SCM). *Biophysik*, **13**, 9.

CERCEK, L. & CERCEK, B. (1976b) Changes in the structuredness of cytoplasmic matrix (SCM) in human lymphocytes induced by PHA and cancer basic protein as measured in single cells. *Br. J. Cancer*, **33**, 539.

CERCEK, L. & CERCEK, B. (1977) Application of the phenomenon of changes in the structuredness of cytoplasmic matrix (SCM) in the diagnosis of malignant disorders: a review. *Eur. J. Cancer*, **13**, 903.

CERCEK, L., CERCEK, B. & FRANKLIN, C. I. V. (1974) Biophysical differentiation between lymphocytes from healthy donors, patients with malignant diseases and other disorders. *Br. J. Cancer*, **29**, 345.

CERCEK, L., CERCEK, B. & OCKEY, C. H. (1973) Structuredness of the cytoplasmic matrix and Michaelis–Menten constants for the hydrolysis of FDA during the cell cycle in Chinese hamster ovary cells. *Biophysik*, **10**, 187.

CURRIE, G. A., HEDLEY, D. W., NYHOLM, R. E. & TAYLOR, S. A. (1978 Contamination of mononuclear cell suspensions obtained from cancer patients by the Böyum method. *Br. J. Cancer*, **38**, 555.

DALE, R. E. & BAUER, R. K. (1971) Concentration depolarization of the fluorescence of dyestuffs in viscous solution. *Acta Physiol. Pol.*, **A40**, 853.

DICKINSON, J. P., DYSON, J. E. D., SMITH, J. J. & COWLEY, N. (1976) The use of a common tumour specific antigen in cancer diagnosis. *Proc. 3rd Int. Congr. Soc. Detection Prevention of Cancer.* New York: Marcel Dekker.

EPSTEIN, M., NORMAN, A., PINKEL, D. & UDKOFF, R. (1977) Flow system fluorescence polarization measurements on fluorescein diacetate-stained EL4 cells. *J. Histochem. Cytochem.*, **25**, 821.

FIELD, E. J. & CASPARY, E. A. (1970) Lymphocyte sensitization: An *in vitro* test for cancer? *Lancet*, **ii**, 1337.

FORRESTER, J. A., DANDO, P. M., SMITH, W. J. & TURBERVILLE, C. (1977) Failure to confirm the macrophage electrophoretic mobility (MEM) test in cancer. *Br. J. Cancer*, **36**, 537.

KREUTZMANN, H., FLIEDNER, T. M., GALLA, H. J. & SACKMANN, E. (1978) Fluorescence polarization changes in mononuclear blood leucocytes after PHA incubation: Differences in cells from patients with and without neoplasia. *Br. J. Cancer*, **37**, 797.

LEWKONIA, R. M., KERR, E. J. L. & IRVINE, W. J. (1973) Clinical evaluation of the macrophage electrophoretic mobility test for cancer. *Clin. Exp. Immunol.*, **14**, 469.

MORLEY, J., WOLSTENCROFT, R. A. & DUMONDE, D. C. (1973) The measurement of lymphokines. In *Handbook of Experimental Immunology 2nd* Edn. Ed. D. M. Weir. Oxford: Blackwell. p. 281.

PICK, E. & TURK, J. L. (1972) The biological activities of soluble lymphocyte products. *Clin. Exp. Immunol.*, **10**, 1.

PREECE, A. W. & LIGHT, P. A. (1974) The macrophage electrophoretic mobility (MEM) test for malignant disease: Further clinical investigations and studies on macrophage slowing factors. *Clin. Exp. Immunol.*, **18**, 543.

PRITCHARD, J. A. V., MOORE, J. L., SUTHERLAND, W. H. & JOSLIN, C. A. F. (1973) Evaluation and development of the macrophage electrophoretic mobility (MEM) test for malignant disease. *Br. J. Cancer*, **27**, 1.

PRITCHARD, J. A. V. & SUTHERLAND, W. H. (1978) Lymphocyte response to antigen stimulation as measured by fluorescence polarization (SCM test). *Br. J. Cancer*, **38**, 339.

PRITCHARD, J. A. V., SUTHERLAND, W. H., SEAMAN, J. E. & 6 others (1978) Cancer-specific density changes in lymphocytes after stimulation with phytohaemagglutinin. *Lancet*, **ii**, 1275.

ROCKLIN, R. E. (1974) Clinical applications of *in vitro* lymphocyte tests. *Prog. Clin. Immunol.*, **2**, 21.

ROTMAN, B. & PAPERMASTER, B. W. (1966) Membrane properties of living mammalian cells as studied by enzymatic hydrolysis of fluorogenic esters. *Proc. Natl Acad. Science*, **55**, 134.

STEEN, H. B. & LINDMO, T. (1976) Membrane transport in lymphocytes *in vitro* studied by a fluoremetric method. *J. Cell Biol.*, **70**, 272a.

TAKAKU, F., YAMANADA, T. & HASHIMOTO, Y. (1977) Usefulness of the SCM test in the diagnosis of gastric cancer. *Br. J. Cancer*, **29**, 345.